# Omega-3 Phospholipids from Krill Oil Enhance Intestinal Fatty Acid Oxidation More Effectively than Omega-3 Triacylglycerols in High-Fat Diet-Fed Obese Mice

**DOI:** 10.3390/nu12072037

**Published:** 2020-07-09

**Authors:** Petra Kroupova, Evert M. van Schothorst, Jaap Keijer, Annelies Bunschoten, Martin Vodicka, Ilaria Irodenko, Marina Oseeva, Petr Zacek, Jan Kopecky, Martin Rossmeisl, Olga Horakova

**Affiliations:** 1 Laboratory of Adipose Tissue Biology, Institute of Physiology of the Czech Academy of Sciences, Videnska 1083, 14220 Prague, Czech Republic; petra.kroupova@fgu.cas.cz (P.K.); ilaria.irodenko@fgu.cas.cz (I.I.); marina.oseeva@fgu.cas.cz (M.O.); jan.kopecky@fgu.cas.cz (J.K.); 2 Human and Animal Physiology, Wageningen University, 6708 WD Wageningen, The Netherlands; evert.vanschothorst@wur.nl (E.M.v.S.); jaap.keijer@wur.nl (J.K.); annelies.bunschoten@wur.nl (A.B.); 3 Laboratory of Epithelial Physiology, Institute of Physiology of the Czech Academy of Sciences, 14220 Prague, Czech Republic; martin.vodicka@fgu.cas.cz; 4 Proteomics Core Facility, Faculty of Science, Charles University, Division BIOCEV, 25250 Vestec, Czech Republic; zacek@natur.cuni.cz

**Keywords:** krill oil, Omega-3 phospholipids, high-fat diet, Omega-3 index, small intestine

## Abstract

Antisteatotic effects of omega-3 fatty acids (Omega-3) in obese rodents seem to vary depending on the lipid form of their administration. Whether these effects could reflect changes in intestinal metabolism is unknown. Here, we compare Omega-3-containing phospholipids (krill oil; ω3PL-H) and triacylglycerols (ω3TG) in terms of their effects on morphology, gene expression and fatty acid (FA) oxidation in the small intestine. Male C57BL/6N mice were fed for 8 weeks with a high-fat diet (HFD) alone or supplemented with 30 mg/g diet of ω3TG or ω3PL-H. Omega-3 index, reflecting the bioavailability of Omega-3, reached 12.5% and 7.5% in the ω3PL-H and ω3TG groups, respectively. Compared to HFD mice, ω3PL-H but not ω3TG animals had lower body weight gain (−40%), mesenteric adipose tissue (−43%), and hepatic lipid content (−64%). The highest number and expression level of regulated intestinal genes was observed in ω3PL-H mice. The expression of FA ω-oxidation genes was enhanced in both Omega-3-supplemented groups, but gene expression within the FA β-oxidation pathway and functional palmitate oxidation in the proximal ileum was significantly increased only in ω3PL-H mice. In conclusion, enhanced intestinal FA oxidation could contribute to the strong antisteatotic effects of Omega-3 when administered as phospholipids to dietary obese mice.

## 1. Introduction

Obesity, i.e., excessive accumulation of white adipose tissue (WAT) in the body, is associated with insulin resistance and metabolic disorders (i.e., “metabolic syndrome”), which in turn increase the risk of type 2 diabetes, cardiovascular disease and premature death. While effective pharmacological interventions for the treatment of metabolic consequences of obesity require the use of multiple agents and are often associated with adverse side effects, lifestyle changes remain an essential component of any prevention or treatment strategy. For instance, increased physical activity and dietary changes could lower the incidence of type 2 diabetes in subjects with elevated fasting glucose concentrations and impaired glucose tolerance by as much as 60% [1].

In terms of the effect of dietary fatty acids (FA) on metabolism, supplementation with polyunsaturated FA of n-3 series (Omega-3) such as eicosapentaenoic acid (EPA; 20:5n-3) and docosahexaenoic acid (DHA; 22:6n-3), which are found in marine fish and fish oils, could reduce the risk of cardiovascular disease [2]. Several studies in obese humans also demonstrated a reduction of adiposity after Omega-3 supplementation [3,4], while Omega-3 prevented the development of obesity, insulin resistance, and dyslipidemia in rodents fed a high-fat diet [5,6,7,8,9]. Moreover, Omega-3 may reduce liver steatosis ([5,10], and reviewed in [11]). Beneficial effects of Omega-3 on metabolism mainly involve lipid-lowering and anti-inflammatory mechanisms [12]. While the molecular targets of Omega-3 in liver [8], WAT [5,6,9] or muscle [7,13] have been intensively studied, the contribution of other organs to the overall metabolic effect of Omega-3 is still little characterized. Previous studies suggest that dietary interventions with Omega-3 supplemented in the form of triacylglycerols (TAG) may also affect intestinal metabolism [14,15]. For instance, fish oil supplementation (i.e., Omega-3 as TAG) induced dose-dependent expression of genes involved in lipid metabolism and FA oxidation in the small intestine but not in the colon of C57BL/6 mice fed a high-fat diet [14]. Enhancement of FA oxidation in the intestine has been shown to protect mice from visceral fat accumulation and impaired glucose homeostasis induced by high-fat feeding [16]. These data suggest that the small intestine may contribute significantly to the metabolic, antisteatotic and possibly also the anti-obesity effects of Omega-3 administration.

Further evidence suggests that the efficacy of Omega-3 in modulating metabolism may depend on the lipid class in which these FA are delivered into the organism. In statin-treated dyslipidemic subjects, EPA and DHA administered as TAG showed a stronger hypolipidemic effect as compared to their ethyl ester form [17]. The superior effect of Omega-3 given as TAG might be attributable to a better bioavailability of EPA and DHA, determined as a relative percentage of EPA and DHA in plasma phospholipids (PL) or red blood cell (RBC) membranes (i.e., Omega-3 index [18]). Moreover, administration of a single dose of EPA and DHA to healthy young men either as re-esterified TAG, ethyl esters or marine PLs resulted in an increase in the Omega-3 index with the following efficacy: PL > TAG > ethyl esters [19]. Importantly, improved bioavailability of DHA and especially EPA in plasma following Omega-3 administration as PL was demonstrated in both human subjects [19,20] and laboratory rodents [21]. In line with improved EPA and DHA bioavailability, Omega-3 PL were able to alleviate many aspects of the metabolic syndrome including dyslipidemia, impaired glucose homeostasis, adipocyte hypertrophy as well as hepatic steatosis in rodents fed a high-fat diet [10,21,22,23]. Importantly, administration of Omega-3 PL contained in krill oil or herring meal extract resulted in a more effective reduction of ectopic fat accumulation in the livers of obese animals, compared to Omega-3 TAG [10,21,24]. Reduction of lipid content in the liver due to administration of Omega-3 PL was associated with strong suppression of gene expression in the de novo FA synthesis and cholesterol biosynthesis pathways [10,22]. However, despite strong evidence for the beneficial effects of Omega-3 PL on metabolism and fat accumulation, the potential role of the small intestine and its metabolism in these effects has not yet been addressed.

Given the available evidence for stimulation of FA catabolism in the small intestine in response to Omega-3 supplementation [14], we hypothesized that the potent antisteatotic effects of Omega-3 PL supplementation, often observed in preclinical models of obesity, may also be related to more effective regulation of intestinal lipid metabolism. Therefore, in this study, we determined the global changes in gene expression as well as FA oxidation in the small intestine of dietary obese mice supplemented with EPA and DHA administered as Omega-3 PL (in the form of krill oil), and compared them with those elicited by the same dose of EPA and DHA but given as TAG.

## 2. Materials and Methods

### 2.1. Animals and Diets

Male C57BL/6N mice (Charles River Laboratories, Sulzfeld, Germany) at the age of 12 weeks were fed a standard low-fat diet (Chow; 3.4% wt/wt as lipids; Rat/Mouse—Maintenance extrudate; Ssniff Spezialdieten GmbH, Soest, Germany). After one week of adaptation to a corn oil-based high-fat diet [HFD; lipids ~35% (wt/wt)], mice were randomly assigned to one of the four experimental diets: (i) HFD; (ii) HFD-based diet, in which 15% (wt/wt) of dietary lipids was replaced (at the expense of the corn oil component) by the TAG-based EPA and DHA concentrate Epax 1050 TG (ω3TG diet; Epax 1050 TG contained ~11% EPA and ~47% DHA (wt/wt); Epax, Ålesund, Norway) to achieve a total EPA and DHA concentration of ~30 mg/g diet (tocopherol content ~0.02% (wt/wt]; (iii) HFD-based diet, in which 45% (wt/wt) of dietary lipids was replaced by PL-based EPA and DHA in the form of Antarctic krill oil (ω3PL-H diet; krill oil contained ~13% EPA and ~7% DHA (wt/wt); Rimfrost USA, Merry Hill, NC, USA) to achieve a total EPA and DHA concentration of ~30 mg/g diet (astaxanthin content ~0.005% (wt/wt)); and (iv) HFD-based diet, in which 15% (wt/wt) of dietary lipids was replaced by Antarctic krill oil (ω3PL-L diet), this time at a dose corresponding to ~10 mg EPA and DHA per g diet (astaxanthin content ~0.002% (wt/wt); see Supplementary Materials Table S1 for macronutrient composition of the diets). Chow-fed mice served as lean controls. The mice were kept in controlled conditions (i.e., 22 °C; 50% humidity; 12 h/12 h light/dark cycle) with free access to food and water. Body weight and 24-h food consumption were recorded every week. Mice were fed the respective diets for 8 weeks and then sacrificed by cervical dislocation under diethyl ether anesthesia (*n* = 8 per group). Blood was collected into EDTA-coated tubes and plasma obtained by centrifugation. The entire small intestine from the pylorus to the ileocecal valve, as well as liver and skeletal muscle (*m. quadriceps femoris*) were removed and snap frozen in liquid nitrogen. The dissected small intestine, excluding the sample for microarray analysis, was subdivided into several segments, namely the duodenum, proximal and distal jejunum, as well as the proximal and distal ileum. Segments were snap frozen in liquid nitrogen and stored at −80 °C for qPCR. All samples were stored at −80 °C for further analysis. In a second experiment of the same experimental design (*n* = 8 per group), the small intestine was dissected and subdivided into several segments, while the proximal ileum was used for explants to measure FA β-oxidation ex vivo (see Section 2.8) and the proximal jejunum was fixed in 4% paraformaldehyde and stored at −80 °C for histological analysis (see Section 2.5). The experiments followed the guidelines for the use and care of laboratory animals of the Institute of Physiology of the Czech Academy of Sciences and were approved under the protocol no. 81/2016.

### 2.2. Biochemical Analysis of Plasma and Tissue Samples

Plasma levels of (i) TAG and total cholesterol were determined using the colorimetric enzymatic assays from Erba Lachema (Brno, Czech Republic), and (ii) non-esterified FA (NEFA) were assessed with a NEFA-HR(2) kit from Waco Chemicals GmbH (Neuss, Germany). Blood glucose levels in both fasting and *ad libitum* fed mice were measured by OneTouch Ultra glucometers (LifeScan, Milpitas, CA, USA).

Liver and muscle TAG content was estimated in ethanolic KOH tissue solubilisates as before [25]. Briefly, tissue samples (~50 mg) were digested with 150 mL of 3 M KOH in 65% ethanol at 70°C for 2 h. Resulting homogenates were cleared from debris by a brief centrifugation and the concentration of total glycerolipids was assessed by the Bio-La-Test TG L250S (Erba Lachema, Brno, Czech Republic).

### 2.3. Oral Glucose Tolerance Test

One week before the end of dietary intervention (i.e., at week 7), oral glucose tolerance test (GTT) was performed after an overnight fast (~15–16 h), as described earlier [26]. A bolus of 200 mg of glucose was administered orally by gavage and blood glucose levels were measured using Contour Plus glucometers (Bayer, Leverkusen, Germany) at time 0 (i.e., before injection) and 15, 30, 60, 120 and 180 min after the gavage. Results were expressed as incremental area under the glucose curve (AUC).

### 2.4. FA Composition in RBC

Extraction of lipids from RBC, subsequent extraction of FA methyl esters and their analysis using comprehensive two-dimensional gas chromatography with mass detection Pegasus 4D (LECO, St. Joseph, MI, USA) was performed as described earlier [27]. The Omega-3 index was calculated as the sum of EPA and DHA levels divided by the total levels of all FA.

### 2.5. Histology

Villi length, crypt height and the thickness of muscular layer were measured in the proximal jejunum. The frozen jejunum embedded in Tissue-Tek block was cut in 20 µm-slices attached to Superfrost Plus (Thermo Scientific, Waltham, MA, USA) slides and stained using hematoxylin. The above intestinal parameters were measured using a Leica LMD 6000 optical microscope and Leica Microdissection software v 6.5 (Leica Microsystems CMS GmbH, Wetzlar, Germany). At least 10 individual measurements for each parameter were performed for each sample. Two intestinal samples (200 µm apart) were taken and measured from each mouse. The villi length was defined as the length from lamina propria to the top of the villus. The crypts were measured from the bottom to the top of the crypt. All histological analyses were performed by a pathologist blinded to dietary groups.

### 2.6. Transcriptome Analysis of the Small Intestine

Total RNA was isolated from small intestine samples using TRI Reagent (Sigma-Aldrich, Prague, Czech Republic) and purified using RNeasy columns (Qiagen, Venlo, The Netherlands). 200 ng RNA of each sample (*n* = 8 per group) was assayed using the Agilent’s Whole Mouse Genome 8 × 60 K Oligo microarrays (Agilent Technologies Inc., Santa Clara, CA, USA), as earlier [10]. All microarray data are available at Gene Expression Omnibus (GEO; GSE93151). Pathway analysis using all differentially expressed genes (DEG) with *p* < 0.05 was performed using the DAVID database [28]. Statistical analysis was performed by a standard procedure using log 2 normalized data.

### 2.7. Real-time Quantitative PCR (RT-qPCR)

To confirm the results of microarray analysis, RT-qPCR was performed as described [9]. Data were normalized to the level of villin expression. An overview of the primer sequences is given in Table S2.

### 2.8. Fatty Acid Oxidation

The level of mitochondrial FA β-oxidation in the intestine (i.e., proximal ileum) was measured as before [14].

### 2.9. Statistical Analysis

Data are presented as means ± SEM. Statistical analysis was performed using SigmaStat software v. 4.0 (Systat Software Inc., San Jose, CA, USA). Data were analysed by two-tailed Student’s *t*-test or one-way ANOVA followed by the Holm-Sidak test. Specific *p* values are stated in the Results section and the legends to figures. A *p* < 0.05 was considered significant.

## 3. Results

### 3.1. Parameters of Energy Balance and Adiposity

**Figure 1:**
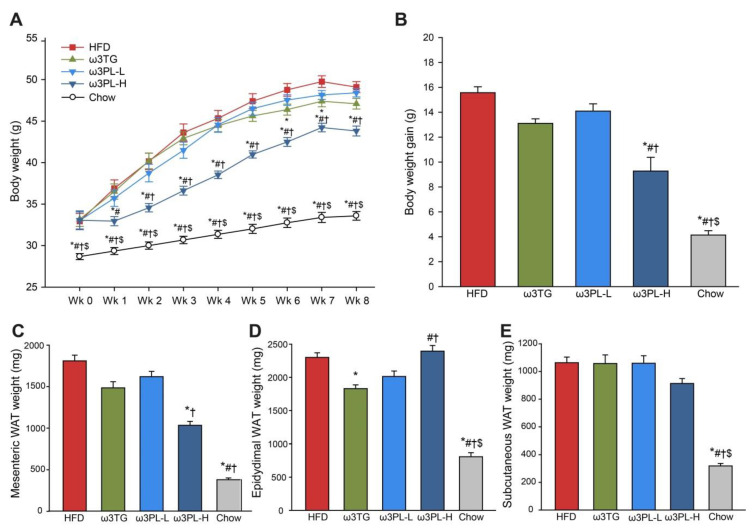
The effect of high-fat diet (HFD) feeding and its supplementation by Omega-3 on body weight (**A**), body weight gain (**B**), mesenteric WAT (**C**), epididymal WAT (**D**) and subcutaneous WAT (**E**) in mice. Tissues were weighed after 8 weeks of dietary intervention, while body weight gain was calculated as difference between Week 7 and 0. Data are means ± SEM (*n* = 8). *, significantly different vs. HFD; #, significantly different vs. ω3TG; †, significantly different vs. ω3PL-L, $, significantly different vs. ω3PL-H. (*p* < 0.05, one-way ANOVA).

First, we characterized the ability of Omega-3 administered as either krill oil (ω3PL-H and ω3PL-L diet) or TAG concentrate (ω3TG diet) to prevent the development of HFD-induced obesity and metabolic disorders. As expected, the rate of body weight gain (Figure 1A), as well as the final weight gain (Figure 1B), were markedly reduced in the Chow-fed mice as compared to HFD group.

Among the groups fed HFD-based diets, ω3PL-H mice had significantly lower body weight compared to other groups of mice, starting from week 1 (week 2 in the ω3PL-L group) until the end of the study (Figure 1A). In addition, both ω3PL-H and ω3TG mice exhibited a reduced cumulative food intake of ~10% during the first 7 weeks of the study (Table 1). To determine to what extent HFD-induced weight gain reflected an increase in adiposity, the weights of various fat depots were analyzed. As expected, at the end of the 8-week dietary intervention period all analyzed fat depots were significantly larger in animals fed HFD-based diets than in lean chow-fed mice (Figure 1C–E and Table 1), with the largest difference observed in the case of mesenteric WAT, showing a ~5-fold increase. Only ω3TG and ω3PL-H diets with higher Omega-3 content were able to reduce the weight of some of the visceral WAT depot, as compared to obese HFD-fed controls (Figure 1C, D). However, there was a depot-specific effect of different lipid classes used to administer Omega-3; while the ω3TG group exhibited reduced epididymal and retroperitoneal WAT by 20 and 23%, respectively (Figure 1D and Table 1), ω3PL-H mice showed a preferential reduction in mesenteric WAT by 43% (Figure 1C). Besides the reduced weight of mesenteric WAT, ω3PL-H mice also had lower liver weight (by 17%; Table 1).

**Figure 2:**
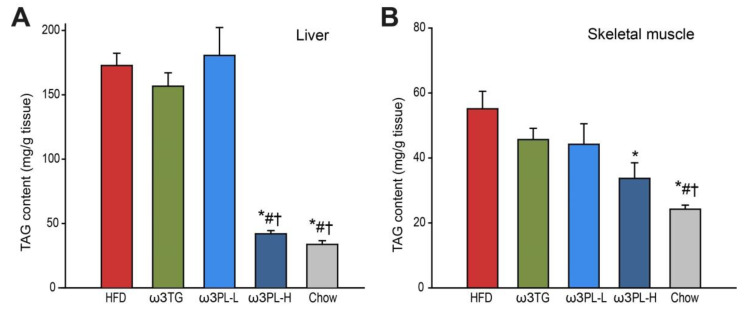
The effect of long-term Omega-3 supplementation on ectopic fat accumulation in dietary obese mice. After 8 weeks of dietary interventions, the level of TAG accumulation was assessed in the liver (**A**) and skeletal muscle (**B**). Data are means ± SEM (*n* = 8). *, significantly different vs. HFD; #, significantly different vs. ω3TG; †, significantly different vs. ω3PL-L, (*p* < 0.05, one-way ANOVA).

To examine the effects of HFD feeding and Omega-3 supplementation in terms of induction or prevention of ectopic lipid storage, we measured the TAG content in tissues such as the liver and skeletal muscle (Figure 2). In the liver (Figure 2A), the TAG content was ~3-fold higher in HFD mice than in the chow-fed animals. While the level of hepatic lipid accumulation in ω3TG and ω3PL-L mice was comparable to HFD-fed controls, ω3PL-H mice were completely protected against hepatic steatosis (Figure 2A). Similar effects of krill oil supplementation (i.e., ω3PL-H) were observed also in the skeletal muscle (Figure 2B).

### 3.2. Effect of Omega-3 Supplementation on Lipid and Glucose Homeostasis

To evaluate potential changes in systemic lipid metabolism due to Omega-3 supplementation, we measured plasma levels of TAG, NEFA and total cholesterol in the fasted state (Table 1). HFD feeding significantly impaired the fasting-induced increase in NEFA plasma levels, while Omega-3 supplementation in ω3PL-H mice normalized this defect (Table 1). Plasma cholesterol levels were increased ~2-fold in HFD mice as compared to lean Chow-fed animals; Omega-3 supplementation in either ω3PL-H or ω3TG mice partially normalized plasma cholesterol levels (Table 1). Despite the fact that plasma levels of TAG did not generally differ between the chow and HFD groups, they were partially increased in both krill oil-supplemented groups and unchanged in ω3TG mice (Table 1).

While HFD feeding substantially increased both fasting blood glucose and plasma insulin levels, the latter parameter was reduced by ~50% in ω3PL-H mice compared to HFD-fed controls (Table 1). Omega-3 supplementation did not change fasting plasma glucose level (Table 1). Furthermore, in ω3PL-H animals, glucose clearance during oral GTT was significantly faster than in other groups of mice fed HFD-based diets, and thus the level of glucose intolerance expressed as AUC was markedly reduced (Table 1).

### 3.3. Lipidomic Analysis of RBC and the Omega-3 Index

**Figure 3:**
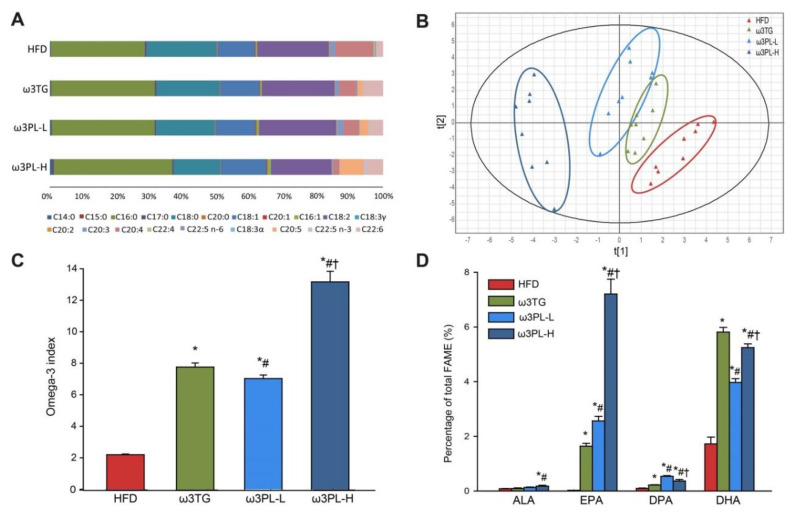
The long-term Omega-3 dietary supplementation affected FA composition of cell membranes. Distribution of FA in the phospholipid fraction of RBCs (**A**) and score plot as assessed by partial-least-squares discriminant analysis (PLS-DA) based on the FA composition in total phospholipids of RBC (**B**) from mice after 8 weeks of dietary intervention. Incorporation of Omega-3 in the RBC membranes expressed as the Omega-3 index (**C**) or relative content of individual Omega-3, ALA, EPA, DPA and DHA (**D**). Data are mean percentage of total FA in phospholipid fraction (**A**) or mean percentage ± SEM (**C**,**D**) (*n* = 8). *, significantly different vs. HFD; #, significantly different vs. ω3TG; †, significantly different vs. ω3PL-L (*p* < 0.05, one-way ANOVA). For detailed FA composition of RBCs phospholipids including Chow-fed mice, see Table S3.

Next, lipidomic analysis of FAs contained in RBC PLs was performed in order to determine the Omega-3 index. The analysis revealed distinct changes in FA profiles as a result of various dietary interventions (Figure 3A and Table S3). Thus, HFD feeding led to a reduction in oleic acid (18:1) and palmitoleic acid (POA; 16:1), as well as to specific changes in the content of saturated FAs (SFA), including palmitic acid (16:0) reduction and increase in stearic acid (18:0) content, which resulted in an unchanged percentage of total SFA as compared to Chow (Table S3). The total content of Omega-6 and Omega-3 FAs remained unchanged in HFD mice. Omega-3, regardless of the form of supplementation, decreased the percentage of total Omega-6, while increasing the percentage of total SFA only in the ω3PL-H group and the percentage of POA in both krill oil-supplemented groups (Table S3).

To characterize in detail the impact of different lipid classes used to administer Omega-3, we analyzed the data on FAs composition in RBC by partial-least-squares discriminant analysis (PLS-DA). When only mice fed HFD-based diets were considered, PLS-DA separated the mice into distinct groups (Figure 3B), with DHA (C22:6), arachidonic acid (AA; C20:4), dihomo-γ-linolenic acid (C20:3), margaric acid (C17:0) and docosapentaenoic acid (DPA; C22:5) identified as the most discriminant metabolites (data not shown).

We then calculated the Omega-3 index, defined as the sum of EPA and DHA in RBC expressed as a percentage of total FAs. As expected, it was increased due to Omega-3 supplementation in the lipid-form-dependent manner, from 2% in HFD mice to 7.5% and 12.5% in the ω3TG and ω3PL-H group, respectively (Figure 3C). Moreover, in ω3PL-L mice the Omega-3 index increased to 6.5%, similar to ω3TG mice whose diet contained three times more EPA and DHA (Figure 3C). Upon close inspection, EPA content in RBC PLs increased from 0.03% in HFD mice to 1.6% and 2.6% in ω3TG and ω3PL-L, respectively, while in the ω3PL-H group EPA content increased to 7.2% (Figure 3D and Table S3). Furthermore, DHA incorporation in RBC PLs increased from 2% in HFD mice to 4.0%, 5.3% and 5.8% in ω3PL-L, ω3PL-H and ω3TG mice, respectively (Figure 3D and Table S3). The content of α-linolenic acid (ALA; C18:3), an essential FA that may serve as a precursor for longer-chain Omega-3, was significantly increased only in ω3PL-H mice (Figure 3D and Table S3), while DPA content increased from 0.11% in HFD fed animals to 0.22%, 0.37% and 0.54% in ω3TG, ω3PL-L and ω3PL-H mice, respectively (Figure 3D and Table S3).

As expected, correlation analysis using pooled data from all Omega-3-supplemented mice suggested an inverse relationship between the level of Omega-3 index and the hepatic TAG content (r = −0.80, *p* < 0.0001; Figure S1A).

### 3.4. The Effect of Omega-3 Supplementation on Intestinal Morphology

**Figure 4:**
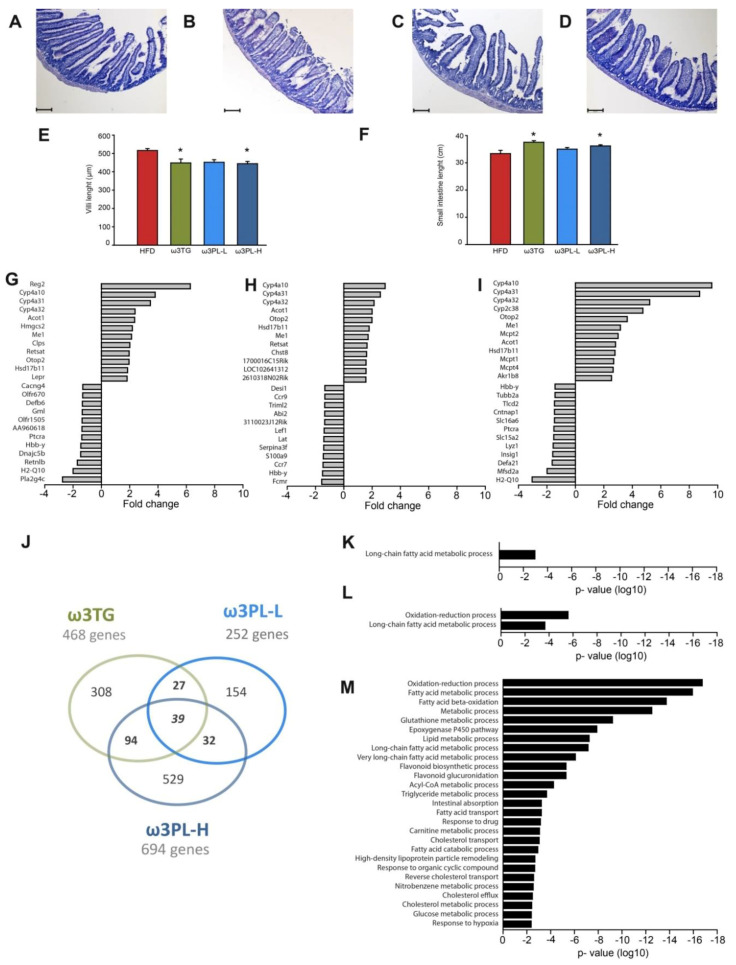
Small intestine as target of Omega-3 dietary intervention in dietary obese mice. (**A**–**D**) Representative hematoxylin-eosin sections of proximal ileum from HFD (**A**), ω3TG (**B**), ω3PL-L (**C**) and ω3 PL-H (**D**) mice; original magnification, 100x; scale bar, 200 µm (**E**) Length of villi in proximal ileum and (**F**) length of small intestine of mice fed HFD with and without supplementation for 8 weeks. Data are means ± SEM (*n* = 8). *, significantly different vs. HFD; #, significantly different vs. ω3TG; †, significantly different vs. ω3PL-L (*p* < 0.05, one-way ANOVA). (**G**–**I**) The 12 most up- and down- regulated genes compared to HFD as assessed by microarray analysis in whole length of small intestine from mice fed ω3TG (**G**), ω3PL-L (**H**) or ω3PL-H (**I**) diet for 8 weeks (*p* < 0.05). (**J**) Venn diagram illustrating the overlap in differentially expressed genes compared to HFD mice as determined by microarray-based RNA analysis in the intestinal samples from ω3TG, ω3PL-L and ω3PL-H mice (*p* < 0.05). (**K**–**M**) Enrichment for Gene Ontology Process terms of genes differentially expressed comparing to HFD in (**K**) ω3TG, (**L**) ω3PL-L and (**M**) ω3PL-H small intestine samples identified by DAVID analysis. Gene Ontology (GO) terms were sorted based on *p*-values (*p* < 0.005). For detailed effects of HFD compared to chow-fed mice, see Figure S2.

As indicated above (see Section 3.1), the abdominal WAT depots are differentially affected by dietary interventions using Omega-3 as TAG or PL. Thus, unlike ω3TG, ω3PL-H feeding resulted in a preferential reduction of mesenteric WAT, which is directly linked to the small intestine. Therefore, we first investigated possible negative changes in intestinal morphology associated with HFD feeding, as well as possible changes associated with Omega-3 supplementation (Figure 4A–D). However, the muscular-serous layer and intestinal crypts in the proximal ileum showed no significant alterations in size or shape between the groups. On the other hand, intestinal villi of the mucosa in both ω3TG and ω3PL-H mice were significantly shorter than those in the control HFD group (Figure 4E); moreover, the small intestine was also significantly longer in the case of ω3PL-H and ω3TG mice (Figure 4F).

### 3.5. Effects of Omega-3 Supplementation on Intestinal Gene Expression

**Figure 5:**
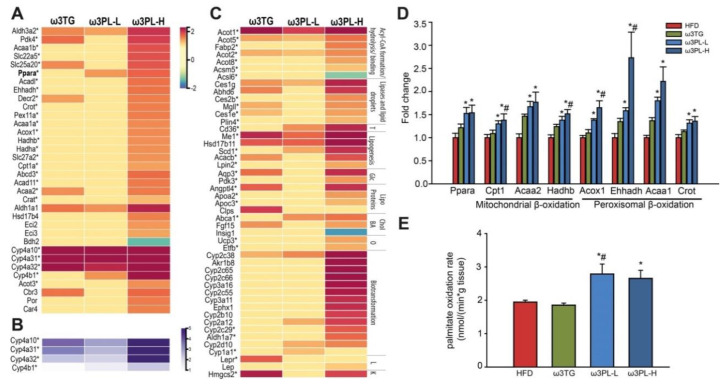
The effect of Omega-3 supplementation on lipid metabolism in the small intestine of dietary obese mice. (**A**) Heatmap showing the gene expression of (**A**) FA oxidation genes, (**B**) ω-oxidation genes (includes also in **A** - for better resolution in different color scale compared to **A**) and (**C**) genes involved in different processes related to lipid metabolism (as indicated on right side) relative to HFD (*p* < 0.05). T, lipid transport; Glc, glucose/glycerol transport and metabolism; Chol BA; metabolism and transport of cholesterol and bile acids; O, oxidative phosphorylation; L, leptin signaling; K, ketogenesis. (**D**) The expression of selected genes of mitochondrial and peroxisomal FA oxidation in proximal ileum. Data were expressed as relative to HFD; HFD = 1. (**E**) Quantification of FA oxidation rate in proximal ileum. Data are means ± SEM (*n* = 8). *, significantly different vs. HFD; #, significantly different vs. ω3TG (*p* < 0.05, one-way ANOVA). For detailed effects of HFD compared to Chow-fed mice, see Figure S2.

To analyze potential differences in the effect of various lipid forms of Omega-3 on the small intestine, we performed whole genome gene expression profiling using the whole small intestine of ω3TG, ω3PL-L and ω3PL-H mice in comparison to the control HFD mice. The highest number of DEG was found in the ω3PL-H group with 694 DEGs, while the expression of 468 and 252 genes was significantly changed in ω3TG and ω3PL-L mice, respectively (Figure 4J). Expression of only a small number of these genes was altered at least two-fold in one or more dietary groups (i.e., 10, 4 and 35 genes in the ω3TG, ω3PL-L and ω3PL-H group, respectively). Moreover, 7 of the 12 most upregulated genes were common to all groups, and included *Cyp4a10*, *Cyp4a31*, *Cyp4a32*, *Otop2*, *Me1*, *Acot1*, and *Hsd17b11*. However, these genes were upregulated to varying degrees, mostly in line with the level of Omega-3 incorporation in each group (Figure 4G–I and Figure 5A–C).

Regenerating islet-derived 2 (*Reg2*), colipase (*Clps*), leptin receptor (*Lepr*) and phospholipase A2 gamma (*Pla2g4c*) were among the most regulated genes in the ω3TG group (Figure 4G and Figure 5A–C), while the ω3PL-L group showed specific up-regulation of a gene related to glycosylation in ER (*Chst8*) and down-regulation of genes related to immune response (e.g., *Fcmr*, *Ccr7*, *Ccr9*, *Lat*, *Lef1*, and *Abl2*; Figure 4H). In contrast, overexpression of mast cells-related genes *Mcpt2*, *1* and *4*, and reduced expression of *H2-Q10*, *Mfsd2a*, *Defa21*, *Insig*, *Lyz1*, *Slc15a2*, *Pctra*, and *Slc16a6* genes were characteristic of ω3PL-H mice (Figure 4I).

### 3.6. Functional Enrichment Analysis of DEGs

Next, we performed functional analysis of gene expression data using DAVID in order to determine which biological processes were affected in the small intestine by exposure to Omega-3 supplementation versus control HFD. All genes showing at least 1.2-fold differential expression were included in the analysis. Functional annotation clustering analysis revealed 1, 2 and 27 significantly affected GO biological processes in the ω3TG, ω3PL-L and ω3PL-H group, respectively (Figure 4K–M). Long-chain FAs metabolism was the only enriched process in ω3TG mice (Figure 4K). Despite the lower number of differentially expressed genes in ω3PL-L compared to ω3TG mice, oxidation-reduction was identified as a highly enriched process, in addition to long-chain FAs metabolism, in these mice (Figure 4L). In ω3PL-H mice the highest number of regulated genes along with the highest level of expression corresponded primarily to 27 affected processes, which included metabolism of FAs, lipids, cholesterol and glucose, as well as intestinal transport and absorption, epoxygenase P450 and glutathione pathways (Figure 4M).

With respect to the effect of Omega-3 supplementation on genes related to FAs and lipid metabolism, the greatest changes were observed in ω3PL-H mice (Figure 5A–C). Specifically, genes belonging to several cytochrome P450 families were among the most upregulated genes in this group of mice. While genes in the CYP4 family, which is involved in FA ω-oxidation, were up-regulated in all Omega-3-supplemented groups (Figure 5A,B), the genes in the CYP2 and CYP3 family, which are capable of catalyzing the oxidative biotransformation of most drugs and other xenobiotics, were upregulated only in ω3PL-H mice (Figure 5C). Furthermore, a high number of genes involved in mitochondrial as well as peroxisomal FA β-oxidation was primarily affected in ω3PL-H mice (Figure 5A), indicating a high level of FA oxidation in the small intestine of these animals. Accordingly, the expression of peroxisome proliferator-activated receptor (PPAR) α, the major transcriptional regulator of FA catabolism, was highest (~1.8-fold increase) in the ω3PL-H group (Figure 5D).

RT-qPCR analysis was used to verify the results of DNA microarrays and to identify possible segment-specific changes in gene expression in the intestine. Eight genes were selected based on microarray analysis, which included the most up-regulated lipid metabolism genes (i.e., *Cyp4a32*, *Me1*, *Acot1*, *Hmgcs2*), genes associated with FA oxidation (*Cpt1*), FAs transport (*Cd36*), lipogenesis (*Scd1*), and finally Pparα transcription factor itself. While the results of RT-PCR analysis were generally in accordance with the data obtained by DNA microarrays (see Table S4), consensus was particularly pronounced in the case of the proximal ileum (Figure 5D). Both ω3PL-L and ω3PL-H mice had increased expression of *Pparα* and its target gene *Cpt1* (i.e., the rate-limiting enzyme for mitochondrial FA β-oxidation) in the proximal ileum (Figure 5D). Therefore, we measured the expression of other FA β-oxidation-related genes in the proximal ileum, namely *Hadhb*, *Acox1*, *Ehhadh*, *Acca1*, *Crot* and *Fabp2*. Consistent with *Cpt1* expression, all these genes were up-regulated in both ω3PL-L and ω3PL-H mice, while their expression was much less affected in ω3TG mice (Figure 5D).

We further correlated the expression of selected genes in the proximal ileum with the Omega-3 index in all Omega-3-supplemented mice. Thus, the expression of genes related to FA ω-oxidation (*Me*, *Cyp4a*) strongly and positively correlated with the Omega-3 index (r = 0.84 and 0.85, respectively; *p* < 0.00001; Figure S1B) and EPA (r = 0.86 and 0.87, respectively; *p* < 0.00001; Figure S1B). We further observed positive correlations, especially with EPA but also with the Omega-3 index, in the case of genes involved in peroxisomal FA β-oxidation (for details see Figure S1B). In contrast, the expression of genes related to mitochondrial FA β-oxidation as well as *Ppara* itself showed only a weak association with EPA or Omega-3 index (for details see Figure S1B). 

### 3.7. Effects of Omega-3 Supplementation on Intestinal Fatty Acid Oxidation

To link up-regulation of FA oxidation genes with an increased pathway flux at the functional level, we measured the rate of FA oxidation ex vivo using proximal ileum explants and ^14^C-palmitate as substrate. Explants from ω3PL-L and ω3PL-H mice had a higher ability to oxidize palmitate (1114 ± 120 and 1062 ± 97 nmol × min^−1^ × g^−1^, respectively) as compared to HFD mice (778 ± 23 nmol × min^−1^ × g^−1^). However, administration of Omega-3 in the form of ω3TG diet did not increase palmitate oxidation above that observed in HFD mice (Figure 5E). The above data suggest that activation of the krill oil-induced intestinal FA β-oxidation transcription program is associated with increased in situ oxidation of FAs, which may help prevent weight gain and glucose intolerance in mice fed a HFD supplemented with this type of Omega-3 PL.

## 4. Discussion

In this study using mice fed a HFD, we showed that chronic Omega-3 PLs supplementation in the form of krill oil induced specific changes in the small intestine, which were characterized by marked up-regulation of the expression of lipid metabolism-related genes, especially those encoding enzymes involved in ω- and β- oxidation of FAs. The above changes were most pronounced in the proximal ileum, and were accompanied by increased in situ FA oxidation, regardless of the krill oil dose. Omega-3 supplementation in the form of TAG, providing the same total amount of EPA+DHA as krill oil (ω3PL-H), failed to induce consistent changes in the expression of FA catabolism genes, except for less pronounced modulation of gene expression of enzymes involved in ω-oxidation of FAs. Thus, some of the effects of krill oil in the intestine were unique, and could also contribute to a preferential reduction of mesenteric WAT and hepatic lipid content in krill oil-supplemented mice.

We showed here that Omega-3 PLs supplementation in the form of krill oil induced a higher degree of Omega-3 incorporation in plasma membranes of RBC (i.e., Omega-3 index) compared to Omega-3 administered as TAG, despite the same EPA+DHA content in the diets. In a previous study, feeding mice a high-fat diet supplemented with another type of marine PLs (i.e., from herring) resulted in higher plasma levels of DHA and especially EPA compared to those achieved by Omega-3 supplementation as TAG [21]. Other studies in rodents and obese subjects suggest that the bioavailability of EPA and DHA from krill oil seems to be higher compared to fish oil [29,30]. However, the fact that various Omega-3 concentrates also differ in the EPA:DHA ratio makes the interpretation difficult. A previous study indicated that Omega-3 concentrate containing EPA and DHA at a 1:1 ratio reached the highest level of Omega-3 absorption [31]. Accordingly, similar levels of EPA and DHA in plasma and RBC were achieved when comparing fish and krill oil with similar ratios of EPA and DHA [32,33], indicating that the type of lipid carrier is not the only factor affecting the bioavailability of Omega-3. In the present study, the Omega-3 index in the ω3PL-H group reached 12.5%, which was associated with an improved metabolic profile, particularly in terms of decreased weight gain, reduced lipid accumulation in ectopic, non-adipose, tissues, and improved glucose tolerance. In humans, it has been shown that an Omega-3 index of ≥8% was associated with the greatest cardio-protection, whereas an index of <4% was associated with the least [34]. This is in contrast to our data in the ω3TG or ω3PL-L group, indicating that the Omega-3 index of 7.5% was not associated with a significant improvement in metabolic profile. Obviously, there are differences between rodents and humans regarding optimal levels of the Omega-3 index. In addition, the Omega-3 index increased to 8% due to the use of highly-enriched Omega-3 concentrate, which was associated with a significant reduction in liver fat content in subjects with relatively low levels of hepatic steatosis [35]. In agreement, we observed a strong positive correlation between the Omega-3 index and the lipid content of the liver. At the same time, mice with Omega-3 index higher than 8% showed a reduction of the liver TAG content to the level similar to that observed in Chow-fed mice, so further reduction cannot be expected.

In terms of the effect of Omega-3 supplementation on body weight, our study showed that ω3PL-H significantly reduced body weight (gain), which was accompanied by decreased weights of liver and mesenteric WAT. In contrast, ω3TG supplementation, providing the same amount of EPA and DHA, significantly reduced the weight of epididymal and perirenal WAT. Such findings are consistent with a previous study in rats showing that fish oil administration reduced epididymal and perirenal WAT without affecting body weight [36]. Krill oil supplementation reduced body weight in various animal models of obesity [37,38] while administering krill powder to mildly obese men tended to decrease the amount of visceral fat [39]. Specific reduction of mesenteric WAT in ω3PL-H mice might be associated with improved glucose tolerance in these animals since previously a stronger involvement of mesenteric WAT in insulin resistance compared to other abdominal WAT depots was shown [40]. Moreover, the improved whole-body glucose tolerance in ω3PL-H mice could also be, at least in part, attributed to increased plasma levels of POA, a lipokine known to enhance insulin signaling [41]. Reduction of hepatic steatosis in ω3PL-H mice is likely to be associated with reduced mesenteric WAT only indirectly, through improved insulin sensitivity and modulation of intestinal lipid transport and metabolism. Although mesenteric WAT is anatomically adjacent to the portal vein, it contributes a relatively small percentage of the total amount of FA taken up by the liver [42].

The main focus of our study was on the effects of various lipid forms of Omega-3 supplementation on intestinal morphology and function. We observed the general effect of Omega-3 in preventing the negative influence of HFD feeding on intestinal length and intestinal villi. Feeding a high-fat diet has been shown to shorten the small intestine, increase villi length, and stimulate the absorption and transport of FA and lipoprotein synthesis [43,44,45]. Such an intestinal adaptation to macronutrient availability/digestibility is more common than only for lipids; for instance, a low versus high digestible starch-containing diet showed an increased small intestinal length, suggestive of enhanced digestion and absorption [46]. Moreover, high-fat feeding is known to impair the integrity of the gut in mice, while Omega-3 supplementation maintains intestinal barrier integrity [47]. In this context, we observed that ω3PL-H upregulated genes related to intestinal barrier function (e.g., *Cyp2c* family members) while ω3TG induced leptin-mediated antimicrobial defense that involves Reg peptide and LepR [48]. The improvement in the intestinal barrier observed in Omega-3 supplemented groups of mice may also play a role in reducing hepatic steatosis, while disruption in the gut-liver barrier (aka “leaky gut”) is common in non-alcoholic steatohepatitis [49].

The metabolic effects of Omega-3 are partially mediated by direct modulation of gene expression. Our transcriptomic analysis used to determine the mechanistic basis of the action of Omega-3 in the small intestine revealed that dietary supplementation with krill oil altered the expression of more genes than fish oil supplying similar amounts of Omega-3. These findings are supported by previous studies suggesting similar superior effects of krill oil on the hepatic transcriptome in mice [50] and on gene expression in peripheral blood mononuclear cells in humans [51]. In the present study, ω3PL-induced DEGs in the small intestine were significantly enriched in 27 GO biological processes, including FAs metabolic processes, intestinal absorption, FAs transport, cholesterol transport, HDL remodeling and glucose metabolism, indicating effective adaptation of the small intestine to lipid overload. However, among the most induced DEGs in all Omega-3-supplemented groups were genes involved in FA ω-oxidation. This finding is supported by previous studies [14,15], where ingestion of fish oil increased expression of members of the cytochrome P450 family 4A (CYP4A) in the small intestine after only two weeks. Although significantly increased expression of the malic enzyme suggests induction of lipogenesis, other lipogenic genes were not affected by dietary interventions. Thus, these data support the idea that malic enzyme is the producer of nicotinamide adenine dinucleotide phosphate (NADPH) needed for CYP4A function [52]. Activation of FA ω-oxidation is important in preventing lipotoxicity during periods of substrate overload, e.g., in the case of high-fat feeding or insufficient mitochondrial FA β-oxidation [53,54]. Moreover, members of the CYP4A family can alleviate obesity-related inflammation, mainly through the anabolism and catabolism of critical anti-inflammatory and pro-resolving lipid mediators [55,56]. Interestingly, although CYP4A members are PPARα targets [57], CYP4A expression corresponded rather to differences in Omega-3 index between the groups, but not with the expression of PPARα itself. In fact, except for several genes primarily involved in lipid synthesis and transport (*Cd36*, *Scd1*, and *Abca1*) and mitochondrial FA β-oxidation in proximal ileum (*Cpt1*, *Acaa2* and *Hadhb*), the expression level of most PPARα-regulated DEGs was more dependent on the Omega-3 index achieved than on the expression of PPARα itself. In previous studies, no relationship between the Omega-3 index and the intestine was characterized besides the effect on intestinal microbial diversity [58] and intestinal barrier function [59]. Omega-3-induced changes in microbiome composition observed in humans did not correlate with the Omega-3 content in RBCs [60]. An association between changes in intestinal metabolism induced by Omega-3 supplementation and bioavailability of EPA and DHA in the gastrointestinal tract can be expected. It is known that the relative content of EPA and DHA in RBC (i.e., the Omega-3 index) correlates strongly with their content in the plasma membranes of the small intestine [61]. Thus, in our study also the relative amounts of EPA and DHA in the gastrointestinal tract of mice should reflect the intergroup differences in the Omega-3 index. Here, we observed for the first time a strong positive association between the Omega-3 index and the expression of genes involved in intestinal ω- and peroxisomal β-oxidation of FAs.

Although the changes in gene expression do not indicate significant changes in the expression of entire metabolic pathways, ω3PL-H regulated several key genes that themselves regulate processes such as the absorption, transport, and metabolism of FA and cholesterol in the small intestine. For example, *Cd36*, well known as FA transporter, plays a role in the absorption and chylomicron formation, secretion of intestinal peptides and is important for maintenance of intestinal homeostasis and epithelial barrier integrity [62]. Furthermore, increased expression of the *Hmgcs2* gene can help maintain a mitochondrial acetyl-CoA pool, strongly affected by enhanced FA oxidation, that is regulated by the spillover pathway of ketogenesis [63]. In addition, β-hydroxybutyrate, the main product of ketogenesis, not only contributes to intestinal cell differentiation [64] but may also improve gut lining integrity impaired by HFD feeding [65,66].

While the results of our transcriptomic analysis reflect global changes at the level of the whole intestine (see above), Omega-3 supplementation can be expected to induce specific changes in different intestinal segments. Therefore, we analyzed the expression of PPARα and its regulated genes in four different segments of the small intestine. Surprisingly, in the proximal ileum, PPARα expression was increased to the same extent in both krill oil-supplemented groups, but not in the ω3TG group. Oxidation of FAs in the intestine has been shown to be regulated by PPARα through control of *Cpt1* and *Acox* expression [67], and mRNA and protein levels of these enzymes have been shown to be strongly correlated in a number of cell types [68,69]. Here we show that krill oil supplementation stimulated mitochondrial FA β-oxidation in conjunction with induction of *Cpt1* expression in a segment- and not dose-specific manner. This is in contrast to the genes involved in peroxisomal FA β-oxidation, which were up-regulated by krill oil in a dose-dependent manner. In addition, it has also been shown in the liver that krill oil stimulates the expression of mitochondrial and peroxisomal FA β-oxidation genes more effectively than fish oil [70]. Although previous studies have indicated that intestinal *Cpt1* expression and oxidation of FAs can also be stimulated by fish oil [14,15], the Omega-3 dose used in both studies was much higher than in the current study. Interestingly, EPA, but not DHA, was able to induce *Cpt1* expression in the intestinal Caco-2/TC7 cell line [71] and rat liver parenchymal cells [72]. Since the incorporation of EPA from krill oil, even when administered at the lower dose (ω3PL-L), exceeds the incorporation of EPA from Omega-3 TAG (ω3TG), this may explain the superior effects of krill oil supplementation in stimulating intestinal mitochondrial FA β-oxidation. However, the fact that the correlation between EPA and CPT1 is only modest suggests that in addition to the EPA content there is another unknown regulatory mechanism for mitochondrial FA β-oxidation induced by krill oil supplementation. Krill oil also contains astaxanthin, a powerful antioxidant with great anti-inflammatory activity, previously associated with lowering TAG levels in the liver, which might be the result of increased hepatic FA β-oxidation [73,74]. However, since the long-term treatment of diet-induced obese mice with astaxanthin administered at a dose four times higher than that used in our experiment did not affect the expression of genes related to FA oxidation in the liver [74], it is unlikely that astaxanthin was responsible for stimulating intestinal mitochondrial FA β-oxidation in our present study. It has been shown that high-fat feeding predominantly up-regulated the activity of FA β-oxidation enzymes in obesity-resistant A/J as compared to obesity-prone C57BL/6J mice, suggesting that the inability to catabolize lipids in the small intestine is associated with the development of diet-induced obesity [75]. In our study, krill oil administration stimulated palmitate oxidation in only one of the five small intestine segments analyzed (i.e., proximal ileum). Despite previous reports showing that constitutive up-regulation of FA β-oxidation throughout the intestine but not selectively in the proximal ileum improved whole-body glucose homeostasis [16], we observed improved glucose tolerance only in mice fed the ω3PL-H diet. Thus, it is more likely that segment-specific stimulation of FA oxidation is closely related to the regulation of intestinal TAG transport, as observed after activation of PPARα in the small intestine by fenofibrate [76]. For this reason, we consider the stimulation of FA oxidation in the small intestine together with changes in the absorption, transport and metabolism of FA to be crucial for the antisteatotic effect of krill oil. Based on available evidence from various transgenic mouse models (reviewed in [77]), the PPAR family of nuclear receptors, as well as histone deacetylases, are key regulators of genes for lipid oxidation in enterocytes, and their genetic manipulation has led to reduced weight gain, hepatic steatosis and serum TAG levels, and improved glucose tolerance. Although it is not clear whether similar changes can be achieved in humans, targeting intestinal metabolism by dietary interventions based on the administration of Omega-3, especially those involving krill oil, remains a promising approach to the treatment of metabolic disorders associated with obesity and metabolic syndrome.

## 5. Conclusions

By using gene expression profiling in the small intestine, we provide evidence that Omega-3 supplemented as PLs from krill oil are more effective than Omega-3 administered at the same dose as re-esterified TAG in terms of effects on intestinal gene expression, while also enhancing mitochondrial β-oxidation of FAs. Despite the fact that both forms of Omega-3 supplementation affected pathways involved in ω-oxidation, krill oil supplementation had more pronounced effects on gene expression and its effect was much broader in terms of the number of pathways affected. This effect of krill oil was linked to improved bioavailability of Omega-3, primarily EPA, and may be responsible for superior metabolic effects of marine PLs, especially when the reduction of hepatic steatosis and improved glucose tolerance are concerned. Thus, our results point to the small intestine and gut-mesenteric WAT-liver interaction as one of the major targets of krill oil administration.

## Figures and Tables

**Table 1:** Body mass, tissue weights, tissue TAG content and plasma parameters in mice fed HFD alone or supplemented with Omega-3.

	HFD	ω3TG	ω3PL-L	ω3PL-H	Chow
**Energy balance**					
Body weight initial (g)	32.94 ± 0.93	33.18 ± 0.86	33.02 ± 1.05	33.06 ± 1.13	28.68 ± 0.36 *
Body weight final (g)	49.09 ± 0.66	47.09 ± 0.64	48.41 ± 0.50	43.81 ± 0.60 *^#†^	33.58 ± 0.52 *^#†$^
Cumulative food intake (MJ/animal)	4.32 ± 0.06	3.93 ± 0.05 *	4.27 ± 0.07 ^#^	3.90 ± 0.07 *^†^	3.60 ± 0.06 *^#†$^
**Tissue weight (mg)**					
Liver	2110 ± 151	2293 ± 172	2523 ± 160	1747 ± 84 ^#†^	1504 ± 87 *^#†^
Brown adipose tissue	182 ± 11	150.3 ± 12	192 ± 5 ^#^	174 ± 12	82 ± 6 *^#†$^
Perirenal WAT	1218 ± 83	940 ± 51 *	1052 ± 67	1002 ± 40	206 ± 24 *^#†$^
**Plasma-fasted state**					
NEFA (mmol/L)	0.61 ± 0.03	0.56 ± 0.02	0.65 ± 0.03	0.81 ± 0.03 *^#†^	0.94 ± 0.03 *^#†$^
Cholesterol (mmol/L)	3.72 ± 0.09	3.05 ± 0.07 *	3.93 ± 0.10 ^#^	3.05 ± 0.07 *^†^	1.87 ± 0.06 *^#†$^
TAG (mmol/L)	0.75 ± 0.04	0.86 ± 0.05	0.92 ± 0.05 *	1.08 ± 0.05 *	0.72 ± 0.05 ^†$^
Glucose (mmol/L)	9.16 ± 0.43	8.59 ± 0.53	9.33 ± 0.47	9.25 ± 0.38	5.81 ± 0.34 *^#†$^
Insulin (pmol/L)	2.18 ± 0.32	1.53 ± 0.28	1.49 ± 0.36	0.95 ± 0.20 *	0.15 ± 0.46 *^#†^
**Glucose homeostasis**					
Glucose AUC (mol/L × 180 min)	3232 ± 249	2561 ± 323 *	2324 ± 228 *	949 ± 97 *^#†^	1805 ± 231 *^#$^

Data are means ± SEM (*n* = 8). Cumulative energy intake was assessed during the initial 7-week period of dietary interventions. Plasma parameters were measured after an overnight fast. *, significantly different vs. HFD; ^#^, significantly different vs. ω3TG; ^†^, significantly different vs. ω3PL-L, ^$^, significantly different vs. ω3PL-H (*p* < 0.05, one-way ANOVA).

## Supplementary Materials

The following are available online at https://www.mdpi.com/2072-6643/12/7/2037/s1, Table S1: Macronutrient composition and energy content in the experimental diets, Table S2: Primers used for RT–qPCR, Table S3: Distribution of fatty acids in phospholipid fraction of red blood cells, Table S4: Comparison of quantified mRNA expressions obtained using microarray analysis in the whole length of small intestine and qPCR in specific segments of small intestine, Figure S1: Potential involvement of Omega-3 bioavailability in metabolic effects of Omega-3. Figure S2. The effect of HFD feeding on the small intestine.
