# Supplementary material for: Omega-3 Phospholipids from Krill Oil Enhance Intestinal Fatty Acid Oxidation More Effectively than Omega-3 Triacylglycerols in High-Fat Diet-Fed Obese Mice
Source: Nutrients. 2020 Jul 9;12(7):2037. doi: 10.3390/nu12072037 (PMC7400938; doi:10.3390/nu12072037)
Supplement: Supplementary file 1 [file nutrients-12-02037-s001.pdf]

Table S1

Macronutrient composition and energy content in the experimental diets

	**HFD**	**ω3TG**	**ω3PL-H**	**ω3PL-L**	**Chow**
Macronutrient composition[a]					
Dry matter (g/100 g)	94.6	95.2	93.3	93.6	90.9
Ash (g/ 100 g)	3.7	3.7	5.5	5.9	6.8
Lipid (g/ 100 g)	29.1	30.2	35.5	34.9	2.9
Carbohydrate (g/ 100 g)	35.9	34.0	30.4	30.5	64.3
Protein (g/ 100 g)	16.4	16.2	21.9	22.3	16.9
Energy density (kJ/ 100 g)	20.4	20.6	20.1	20.6	13.0
Supplement					
Epax 1050 TG (g/ 100 g)	0.0	5.3	0.0	0.0	0.0
Krill oil (g/100g)	0.0	0.0	15.4	5.3	0.0
EPA+DHA content (g/ 100g)	0.0	3.3	3.3	1.1	0.0
EPA content (g/ 100g)	0.0	0.8	2.1	0.7	0.0
DHA content (g/ 100g)	0.0	2.5	1.2	0.4	0.0

[a]The macronutrient composition of experimental diets was assessed by drying (Dry matter), gravimetry (Ash), Kjeldahl method (Protein), and the extraction after acid hydrolysis (Lipids), while the concentration of carbohydrates was calculated.

Table S2
Primers used for qRT –PCR

Gene name	Symbol	Genbank Accesion number	1. Forward primer<br>2. Reverse primer
acetyl-Coenzyme A acyltransferase 1A	*Acaa1*	NM_130864.3	AAAGCAGGGCTGACTGTGAA<br>TGAGTAGCGTGACAACCTGC
acetyl-Coenzyme Aacyltransferase 2 (mitochondrial 3-oxoacyl-Coenzyme A thiolase)	*Acaa2*	NM_177470.3	AGAGTCGTGGGCTACTTCGT<br>AGCTTCGTTCACGTCTATCAAA
acyl-Coenzyme A oxidase 1, palmitoyl	*Acox1*	NM_001271898.1	GCTGGGCTGAAGGCTTTTACTACC<br>CACCTGCTGCGGCTGGATAC
carnitine O-octanoyltransferase	*Crot*	NM_023733.3	AAGCCAAAGCCCAACATCTCAA<br>CAGCAACCAGGGCGTCCAT
carnitine palmitoyltransferase 1a, liver	*Cpt1a*	NM_013495.2	GCAGCTCGCACATTACAAGGACAT<br>AGCCCCCGCCACAGGACACATAGT
CD36 molecule	*Cd36*	NM_001159555.1	TGATACTATGCCCGCCTCTCC<br>TTCCCACACTCCTTTCTCCTCTAC
cytochrome P450, family 4, subfamily a, polypeptide 32	*Cyp4a32*	NM_001100181.1	ACTTTCTTCCAATGGCCGCT<br>GTCAGACATGCTGTCCCCAT
enoyl-Coenzyme A, hydratase/ 3-hydroxyacyl Coenzyme A dehydrogenase	*Ehhadh*	NM_023737.3	CCCCAATTGCTGATATGCTCTGTG<br>ATGCGGAATGCCTCGTTGATAAG
fatty acid binding protein 2, intestinal	*Fabp2*	NM_007980.3	CAGTCTAGCAGACGGAACGG<br>CTCCTTCATATGTGTAGGTCTGGA
3-hydroxy-3-methylglutaryl-Coenzyme A synthase 2	*Hmgcs2*	NM_008256.4	GAGGCCTTCAGGGGTCTAAA<br>GGGAGGCCTTGGTCTTCTG
hydroxyacyl-Coenzyme A dehydrogenase/3-ketoacyl-Coenzyme A thiolase/enoyl-Coenzyme A hydratase	*Hadhb*	NM_001289798	GAACACTGGCAAGGCTGGATT<br>CTAGAAGGAGGCGACCGACTGA
malic enzyme 1	*Me1*	NM_001198933.1	TCTCACTGCCCAGGCTACACTAAC<br>CCTCCGTTAGCTTTGTTCTCTTTG
peroxisome proliferator activated receptor alpha	*Ppara*	NM_001113418.1	TGCGCAGCTCGTACAGGTCATCAA<br>CCCCCATTTCGGTAGCAGGTAGTCTTA
stearoyl-Coenzyme A desaturase 1	*Scd1*	NM_009127.4	TAGCTTTGGGTGCCTTATCTCTTTC<br>CTCTCCAGCCAGCCTCTTGACTATTC
vilin 1	*Vil1*	NM_009509.2	GCCAGATTGCTGACGAGGTT<br>CCCAAGGCCCTAGTGAAGTC

Table S3

Distribution of fatty acids in phospholipid fraction of red blood cells

		HFD	ω3TG	ω3PL-L	ω3PL-H	Chow
Myristic acid C14:0	SFA	0.24 ±0.01	0.30±0.02	0.46±0.03[ab]	0.98±0.07[abc]	0.37±0.01 [ad]
Pentadecylic acid C15:0	SFA	0.10 ±0.00	0.12±0.01[a]	0.14±0.00 [a]	0.22±0.01[abc]	0.11±0.01 [cd]
Palmitic acid C16:0	SFA	28.21±0.46	31.07±0.42[a]	30.86±0.55 [a]	35.57±0.72[abc]	34.47±0.46[abc]
Margaric acid C17:0	SFA	0.43±0.01	0.47±0.02[a]	0.37±0.01[ab]	0.37±0.01	0.33±0.01[abc]
Stearic acid C18:0	SFA	21.07 ±0.27	18.92±0.33[a]	17.65±0.31[ab]	14.08±0.22	13.97±0.20[abc]
Arachidic acid C20:0	SFA	0.25±0.02	0.21±0.03	0.24±0.0	0.19±0.01	0.21±0.01
**Saturated fatty acids TOTAL**		50.30±0.60	51.08±0.48	49.71±0.68	51.42±0.66	49.45±0.53
Palmitoleic acid C16:1	ω−7	0.52±0.04	0.52±0.04	0.80±0.04[ab]	1.05±0.08[abc]	2.39±0.12[abcd]
**ω−7 fatty acids TOTAL**		0.52±0.04	0.52±0.04	0.80±0.04[ab]	1.05±0.08[abc]	2.39±0.12[abcd]
Oleic acid C18:1	ω−9	11.14±0.13	11.82±0.19[a]	11.96±0.17 [a]	13.60±0.16[abc]	16.38±0.21[abcd]
Gondoic acid C20:1	ω−9	0.26±0.02	0.21±0.02	0.31±0.02	0.32±0.04	0.43±0.02[ab]
**ω−9 fatty acids TOTAL**		11.40±0.12	12.02±0.20	12.27±0.17	13.92±0.16	16.81±0.22
Linolelaidic acid C18:2	ω−6	21.29±0.38	21.84±0.58	23.14±0.05	18.18±0.77[abc]	15.77±0.32[abcd]
γ-Linolenic acid C18:3	ω−6	0.10±0.01	0.03±0.01[a]	0.06±0.01	0.06±0.01	0.10±0.01 [b]
Eicosadienoic acid C20:2	ω−6	0.27±0.01	0.19±0.01[a]	0.22±0.01	0.09±0.01[abc]	0.18±0.01[ad]
Dihomo-γ-linolenic acid C20:3	ω−6	1.65±0.10	1.07±0.08	1.95±0.16 [b]	0.36±0.02[abc]	1.49±0.07[bcd]
Arachidonic acid C20:4	ω−6	11.58±0.41	5.10±0.23[a]	4.71±0.24 [a]	1.76±0.09[abc]	10.70±0.52[bcd]
Adrenic acid C22:4	ω−6	0.45±0.04	0.03±0.01[a]	0.04±0.01	0.00±0.00 [a]	0.50±0.05[bcd]
Osbond Acid C22:5	ω−6	0.25±0.01	0.35±0.01	0.06±0.06	0.00±0.00 [a]	0.43±0.08 [cd]
**ω−6 fatty acids TOTAL**		35.60±0.52	28.62±0.48[a]	30.19±0.53 [a]	20.45±0.76[abc]	29.45±0.76[ad]
α-Linolenic acid C18:3	ω−3	0.09±0.01	0.09±0.02	0.13±0.02	0.18±0.03	0.12±0.01
Eicosapentaenoic acid C20:5	ω−3	0.03±0.01	1.64±0.11[a]	2.56±0.17[ab]	7.21±0.54[abc]	0.07±0.01[bcd]
Docosapentanoic acid C22:5	ω−3	0.11±0.01	0.22±0.01[a]	0.37±0.05[ab]	0.54±0.03[abc]	0.15±0.01[bcd]
Docosahexaenoic acid C22:6	ω−3	1.95±0.03	5.81±0.17[a]	3.97±0.14[ab]	5.25±0.13[abc]	1.83±0.04[bcd]
**ω−3 fatty acids TOTAL**		2.19±0.04	7.76±0.26[a]	7.03±0.22 [a]	13.17±0.67[abc]	2.18±0.04[bcd]
Omega-6/ Omega-3 ratio		16.26±0.28	3.73±0.18[a]	4.32±0.11[ab]	1.59±0.14[abc]	13.46±0.5[abcd]
Omega-3 index		2.00±0.04	7.45±0.25[a]	6.53±0.2[ab]	12.45±0.66[abc]	1.91±0.04[bcd]

Data are mean percentage of total fatty acids in phospholipid fraction ± SEM (n = 8). a, significantly different vs. HFD; b, significantly different vs. ω3TG; c, significantly different vs. ω3PL-L; d, significantly different vs. ω3PL-H ($p < 0.05$, one-way ANOVA).

Table S4

Comparison of quantified mRNA expressions obtained using microarray analysis in whole lenght of small intestine and qPCR in specific segments of small intestine.

	Microarrays			qRT-PCR												Process
	Whole lenght of small intestine			Duodenum			Proximal jejunum			Distal jejunum			Proximal ileum			
**Gene**	**ω3TG**	**ω3PL-L**	**ω3PL-H**	**ω3TG**	**ω3PL-L**	**ω3PL-H**	**ω3TG**	**ω3PL-L**	**ω3PL-H**	**ω3TG**	**ω3PL-L**	**ω3PL-H**	**ω3TG**	**ω3PL-L**	**ω3PL-H**	
Pparα	1.00	1.48	1.77	1.00	1.00	1.66	1.00	1.00	1.41	1.00	1.00	1.58	1.00	1.53	1.54	
Cpt1	1.00	1.00	1.44	1.65	1.00	1.00	1.00	1.00	1.00	1.00	1.00	1.00	1.00	1.30	1.38	β-oxidation (mitochondrial)
Acot1	2.35	2.00	2.82	2.18	1.82	2.58	1.41	1.00	1.53	1.30	1.00	1.53	2.46	2.53	4.63	β-oxidation
Cyp4a32	2.38	2.14	5.22	2.71	1.97	6.14	2.63	2.05	5.73	1.80	1.51	3.72	4.97	5.96	23.69	ω-oxidation
Cyp4a10	3.80	2.92	9.58													ω-oxidation
Me	2.13	1.71	3.16	1.82	1.37	2.18	1.61	1.00	1.91	1.49	1.31	2.28	2.03	2.23	5.57	NADPH production
Hmgcs2	2.19	1.00	1.96	2.33	1.50	2.23	1.72	1.00	1.00	1.57	1.00	1.53	2.76	2.80	4.06	Ketogenesis
CD36	1.00	1.51	2.10	1.56	1.51	2.10	1.00	1.00	1.41	1.00	1.00	1.38	1.37	1.51	2.10	FA transport
Scd1	1.00	1.39	2.09	1.31	1.00	1.00	-1.45	1.00	1.38	-2.13	1.00	1.95	1.00	2.36	3.06	Lipogenesis

	Microarrays			Duodenum			Proximal jejunum			Distal jejunum			Proximal ileum			Process
**Gene**	**ω3TG**	**ω3PL-L**	**ω3PL-H**	**ω3TG**	**ω3PL-L**	**ω3PL-H**	**ω3TG**	**ω3PL-L**	**ω3PL-H**	**ω3TG**	**ω3PL-L**	**ω3PL-H**	**ω3TG**	**ω3PL-L**	**ω3PL-H**	
Acaa2	1.53	1.00	1.62										1.46	1.67	1.77	β-oxidation (mitochondrial)
Hadhb	1.00	1.00	1.53										1.00	1.38	1.52	β-oxidation (mitochondrial)
Acox1	1.00	1.00	1.53										1.00	1.38	1.65	β-oxidation (peroxisomal)
Ehhadh	1.00	1.00	1.65										1.35	1.62	2.73	β-oxidation (peroxisomal)
Acaa1	1.00	1.00	1.54										1.37	1.80	2.22	β-oxidation (peroxisomal)
Crot	1.00	1.00	1.63										1.00	1.31	1.36	β-oxidation (peroxisomal)
Fabp2	1.00	1.00	1.60										1.00	1.00	1.00	FA interacellular transport

Results are expressed as fold change to HFD (n=8). Only significant gene expression regulations in response to dietary Omega-3 intervention were used. Cyp4a32 and Cyp4a10 were due to similarity indistinguishable when measured using RT-PCR, therefore the results in segments are sum of both of them.

A)

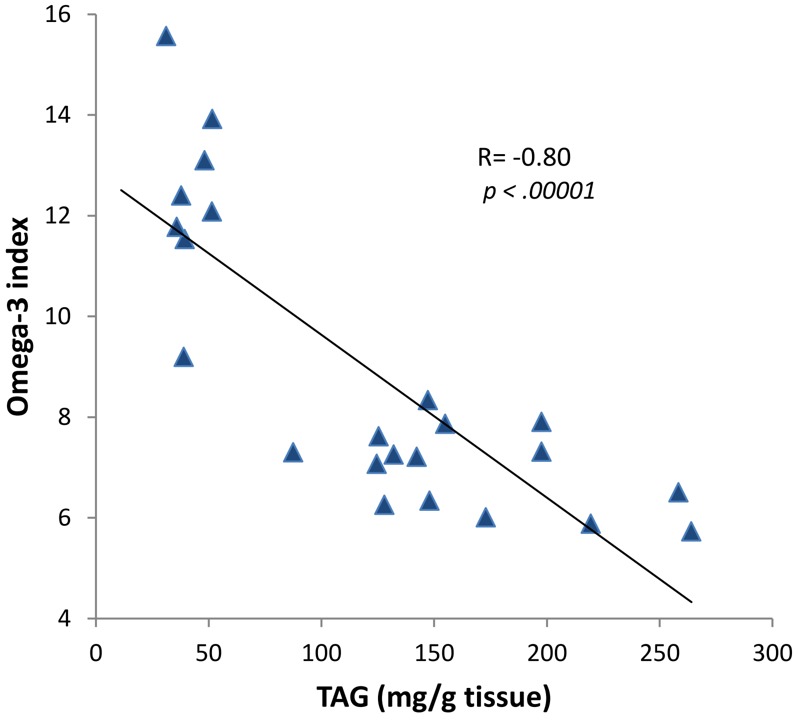

B)

	EPA	*DPA n-3*	*DHA*	*omega-3 index*
PPARα	**0.53**	**0.72**	-0.33	0.39
Cpt1	**0.65**	**0.64**	-0.15	**0.55**
Hadhb	**0.57**	**0.55**	-0.22	**0.46**
Acca2	**0.53**	**0.52**	-0.19	0.44
Acox1	**0.71**	**0.68**	-0.20	**0.60**
Ehhadh	**0.72**	**0.59**	0.06	**0.68**
Acca1	**0.84**	**0.79**	-0.14	**0.73**
Crot	0.28	**0.48**	-0.34	0.16
Me	**0.86**	**0.72**	0.19	**0.84**
Cyp4a	**0.87**	**0.66**	0.18	**0.85**
Hmgcs2	**0.56**	**0.48**	0.07	**0.53**
CD36	**0.65**	**0.47**	0.11	**0.62**
Fabp2	**0.52**	**0.52**	-0.20	0.42
Slc27a2	**0.48**	**0.51**	-0.31	0.36

Figure S1. Potential involvement of Omega-3 bioavailability in metabolic effects of Omega-3. (A) The relationship between Omega-3 index and the TAG content in the liver. (B) Relationships of Omega-3 index and EPA, DPA and DHA bioavailability with gene expression in proximal ileum in mice fed HFD with Omega-3 supplementation for 8 weeks. Pearson correlation coefficient strength as indicated in figure and the table. Bold values in the table denote statistical significance at the $p < 0.05$ level.

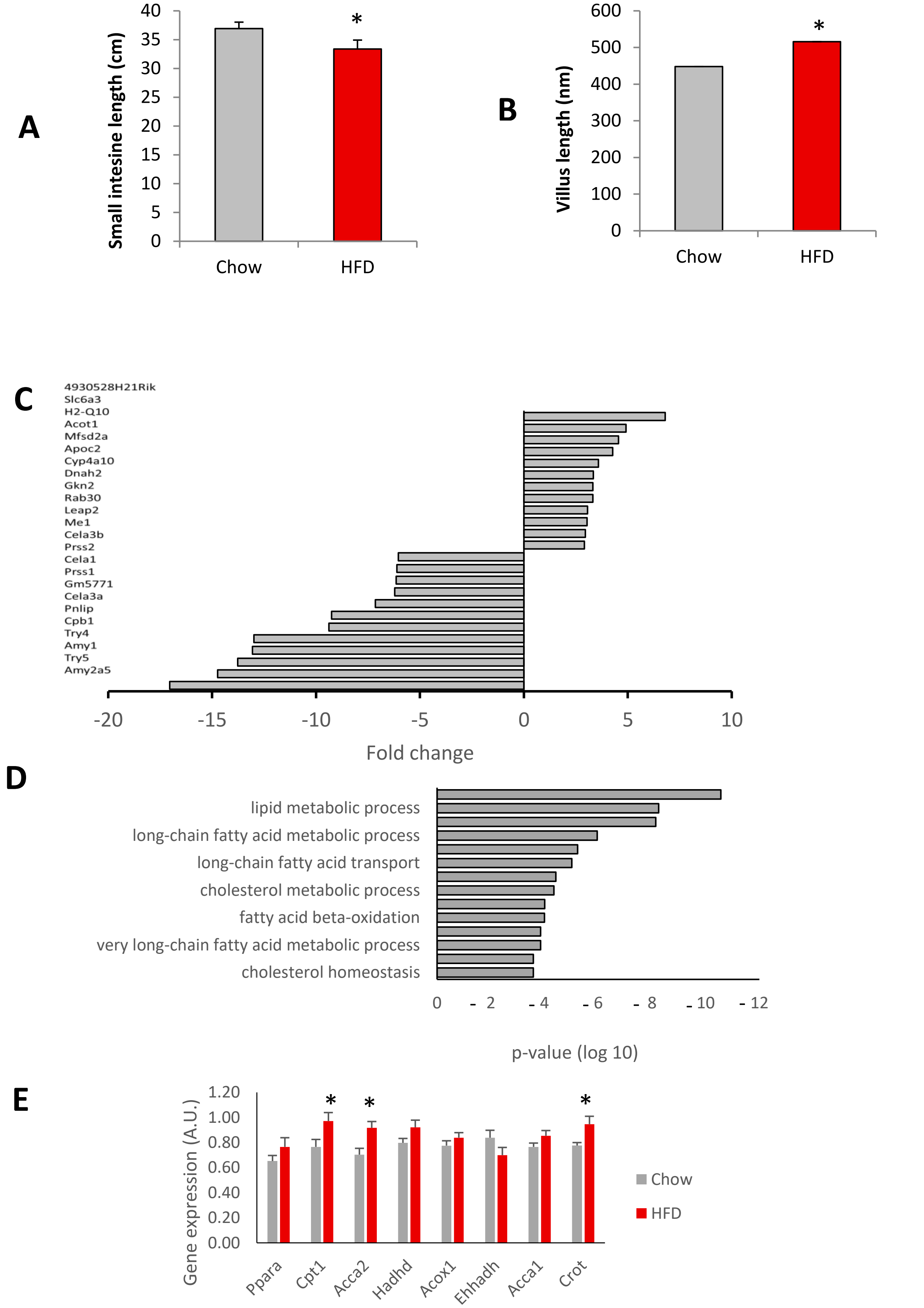

Figure S2. The effect of HFD feeding on the small intestine. (A) Length of small intestine and (B) length of villi in proximal ileum of mice fed Chow or HFD for 8 weeks. (C) The 12 most up- and down regulated genes compared to Chow as assessed by microarray analysis in whole length of small intestine from mice fed HFD. (D) Enrichment for Gene Ontology Process terms of genes differentially expressed between the HFD and Chow groups in the small intestine (identified by DAVID analysis). GO terms were sorted based on p-values ($p < 0.005$). (E) The expression of selected genes of mitochondrial and peroxisomal FA oxidation in proximal ileum. Data are means ± SEM ($n = 8$). *, significantly different vs. Chow (t-test; $p < 0.05$)
